# Supplementary material for: Metastatic Prostate Cancer Cells Secrete Methylglyoxal-Derived MG-H1 to Reprogram Human Osteoblasts into a Dedifferentiated, Malignant-like Phenotype: A Possible Novel Player in Prostate Cancer Bone Metastases
Source: Int J Mol Sci. 2021 Sep 22;22(19):10191. doi: 10.3390/ijms221910191 (PMC8508123; doi:10.3390/ijms221910191)

Supplementary Material: Metastatic prostate cancer cells secrete methylglyoxal-derived MG-H1 to reprogram human osteoblasts into a dedifferentiated, malignant-like phenotype: a possible novel mechanism in prostate cancer bone metastasis

Cinzia Antognelli, Lorella Marinucci, Lara Macchioni, and Vincenzo Nicola Talesa

Figure S1. Level of methylglyoxal (MG)-derived MG-H1 in the fresh medium of osteoblasts (OB) and PC3 cells or in the conditioned medium (CM) from LNCaP cells, derived from a left supraclavicular lymph node metastasis, DU-145 cells, derived from a central nervous system metastasis, and non-cancerous cells PNT2, grown for 24 hours in a humidified atmosphere at 37°C. MG-H1 was evaluated by WB.

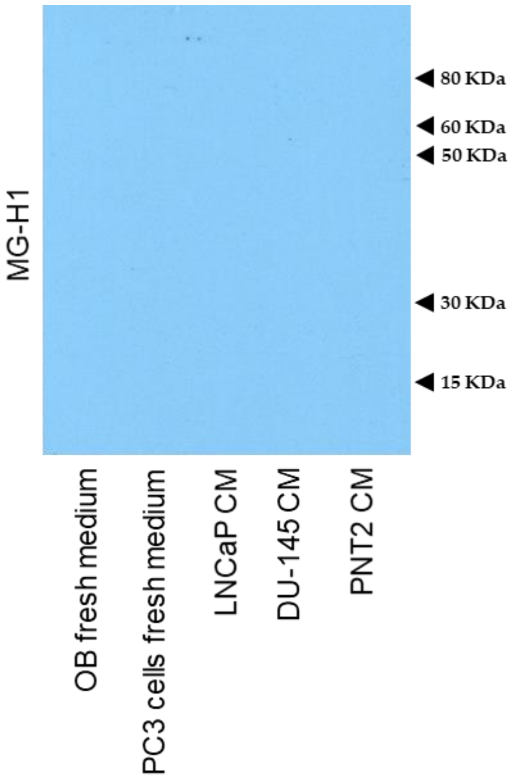

Figure S2. Level of methylglyoxal (MG)-derived MG-H1, measured by a specific ELISA assay, in the conditioned medium (CM) of PC3 cells naïve or pre-treated with the specific MG scavenger aminoguanidine (AG). As shown, AG prevented MG-derived MG-H1 formation and consequent release into the medium. ***p < 0.001

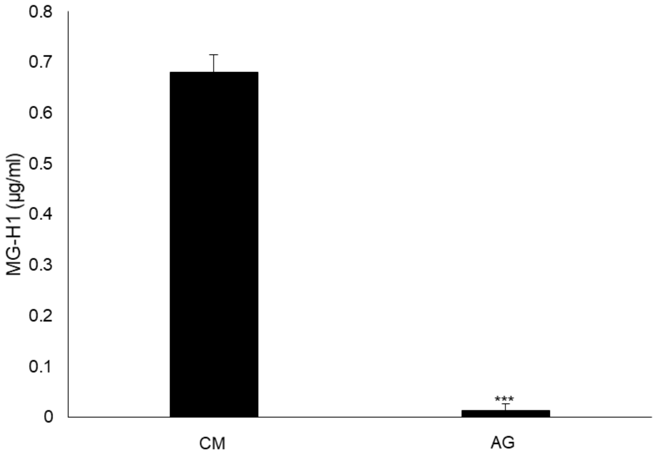

Figure S3. (a) Glyoxalase 1 (Glo1) silencing by small interfering RNA in PC3 cells. Cells were transfected for 72 hours with vehicle alone (Mock), control non-targeting siRNA (siControl, siCtr) and Glo1-siRNA (siGlo1) or non-transfected (NT). Glo1 mRNA expression was detected by qRT-PCR while Glo1 enzymatic specific activity (s.a.) was detected by a spectrophotometric method. (b) Level of methylglyoxal (MG)-derived MG-H1, measured by a specific ELISA assay, in the conditioned medium (CM) of PC3 cells naïve or pre-treated with siCtr or siGlo1 oligonucleotides. mRNA levels, by qRT-PCR, of the mesenchymal trans-differentiation-associated markers Vimentin (VIM) and TGF-β1 (c), cadherin 11 (CDH11) and integrin β1 (ITGB1) (d), the markers associated with OB mature phenotype Runt-related transcription factor 2 (Runx2) and osteonectin (ON) (e) and the pre-osteoblast state marker CD44 and (f) proteins involved in the formation of filopodia-like protrusions Fascin (FASC) and Radixin (RADX) (h) as well as (g) migration and invasion were evaluated in OB exposed for 24 hours to the CM of PC3 cells naïve or pre-treated with siCtr or siGlo1 oligonucleotides. All the data are means ± SD of three independent experiments done in duplicate. *p < 0.05, **p < 0.01, ***p < 0.001 significantly different from untreated control or CM exposed cells.

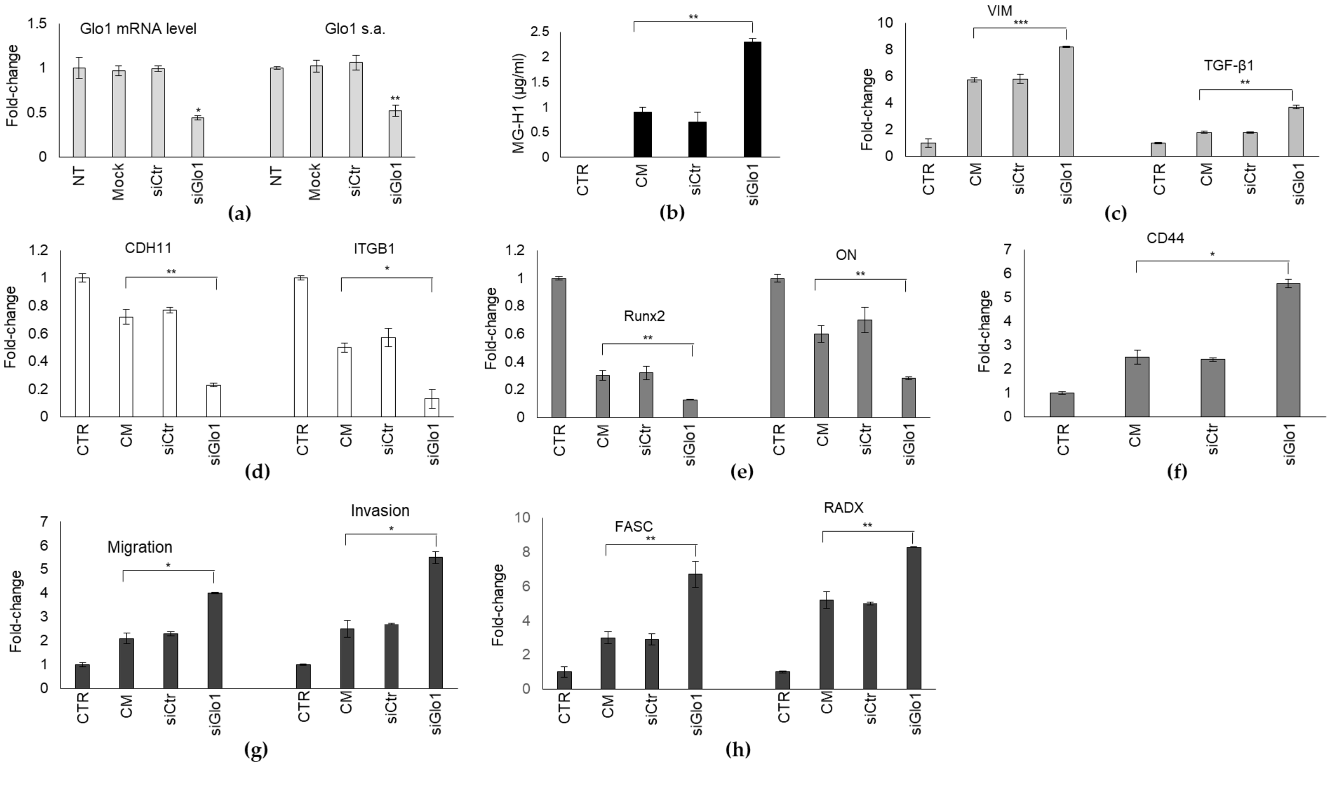

Figure S4. Conditioned medium (CM) from LNCaP cells (LNCaP CM), devoid of MG-H1 (Figure S1), was administrated to human primary osteoblasts (OB) for 24 hours and (a) cell viability (by MTT assay), (b) cell morphology (by light microscopy), transcript levels (by Real-Time PCR) of (c) the mesenchymal trans-differentiation-associated markers vimentin (VIM) and cadherin 11 (CDH11), (d) the markers associated with OB mature phenotype runt-related transcription factor 2 (Runx2) and the pre-osteoblast state marker CD44, (e) migration and invasion (by specific assays), (f) osteocalcin (OC) expression (by Real-Time PCR) and calcium deposits (by alizarin red staining method) (for extracellular matrix mineralization) as well as (g) PSA/PSMA expression (by Real-Time PCR) were evaluated. Control cells (CTR) are represented by OB cultured for the same period of time in their specific growth medium. All the data are means ± SD of three independent experiments done in duplicate. All results did not reach statistical significance ($p > 0.05$ compared with CTR cells).

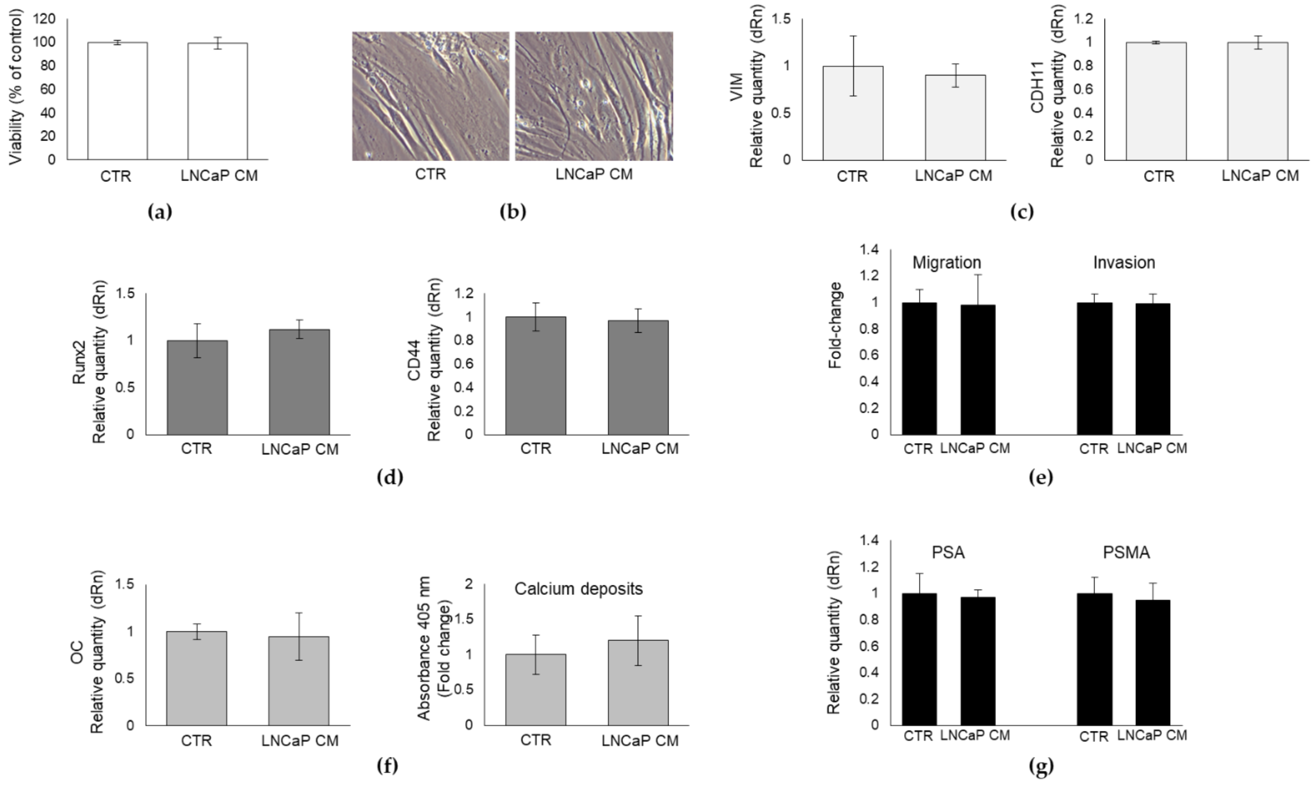

Figure S5. Whole blots reported in Figure 1b, Figure 10a, Figure 11c and Figure S1.

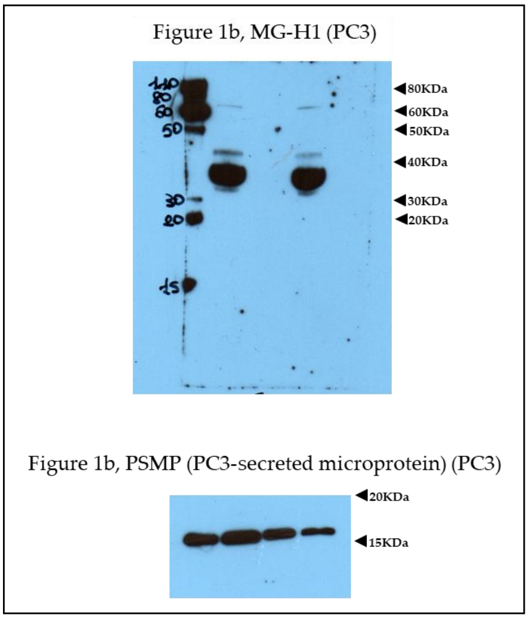

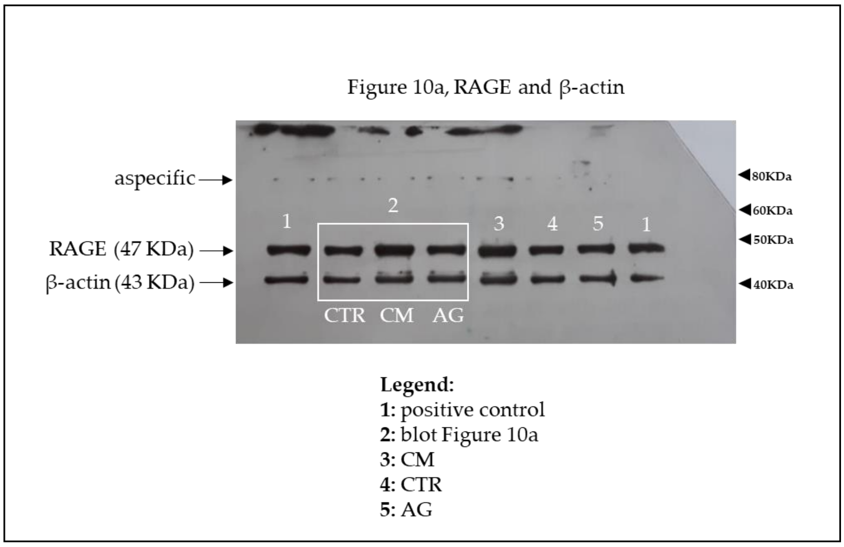

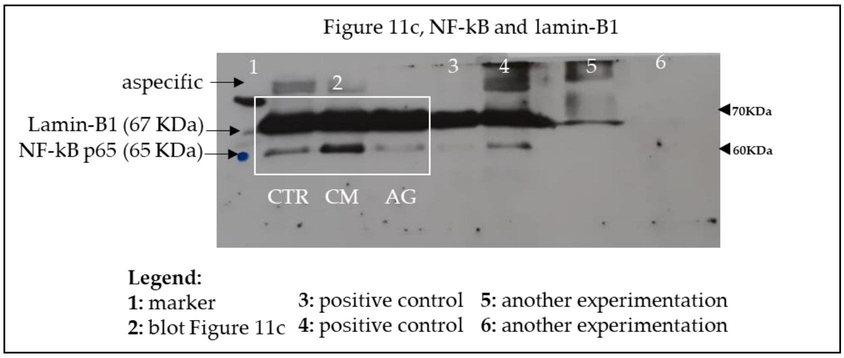

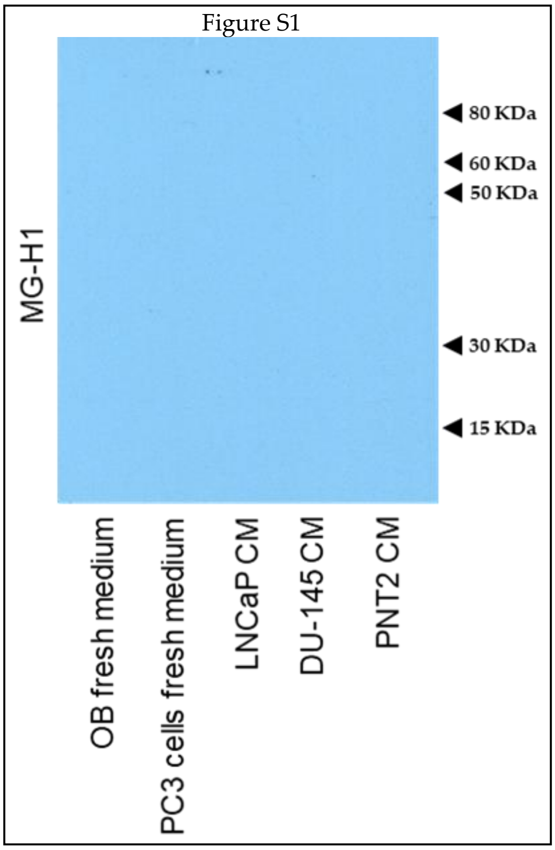

Supplement: Supplementary file 1 [file ijms-22-10191-s001.zip › ijms-1371128-supplementary.pdf]
